# A real-world study of anlotinib combined with GS regimen as first-line treatment for advanced pancreatic cancer

**DOI:** 10.3389/fendo.2023.1110624

**Published:** 2023-01-20

**Authors:** Gouling Zhan, Jianbing Hu, Shijian Da, Jie Weng, Chuanyi Zhou, Fang Wen, Songlian Liu, Fang Fang, Erdong Shen, Qiang Zhou, Pan Luo, Min Xu, Dahe Zhan, Yuqi Su

**Affiliations:** ^1^ Department of Oncology, Yueyang Central Hospital, Yueyang, China; ^2^ Department of Oncology, Third Xiangya Hospital, Central South University, Changsha, China; ^3^ Department of Oncology, Yueyang People’s Hospital, Yueyang, China; ^4^ Department of Hepatobiliary Surgery, Yueyang Central Hospital, Yueyang, China

**Keywords:** pancreatic cancer, anlotinib, GS regimen, efficacy, safety, overall survival, progression-free survival

## Abstract

**Background:**

Anlotinib may boost the efficacy of pancreatic cancer (PC) treatment if timely added to the GS regimen (Gemcitabine, Tegafur-gimeracil-oteracil potassium); however, no data has been published. This study evaluated the safety and efficacy of anlotinib in combination with the GS regimen(hereafter referred to as the A+GS regimen) in the first-line treatment of patients with unresectable or metastatic PC.

**Methods:**

Patients with unresectable or metastatic PC treated at Yueyang Central Hospital and Yueyang People’s Hospital between October 2018 and June 2022 were enrolled in this retrospective real-world investigation. Treatment efficacy was evaluated based on the overall survival (OS), progression-free survival (PFS), disease control rate (DCR), and objective response rate (ORR), while the treatment safety was assessed by the frequency of major adverse events (AEs).

**Results:**

Seventy-one patients were included in this study, 41 in the GS group and 30 in the A+GS group. The A+GS group had a longer mPFS than the GS group (12.0 months (95% CI, 6.0–18.0) and 6.0 months (95% CI, 3.0–8.1)), respectively (P = 0.005). mOS was longer in the GS+A group) when compared with the GS group (17.0 months (95%CI, 14.0–20.0) and 10.0 months (95% CI, 7.5–12.5)), respectively (P = 0.018). The GS+A group had higher ORR (50.0% vs 26.8%, P = 0.045) and DCR (83.3% vs 58.5%, P = 0.026). Furthermore, there were no grade 4-5 AEs and no treatment-related deaths, and no discernible increase in AEs in the GS+A group when compared with the GS group.

**Conclusion:**

The A+GS regimen therapy holds great promise in managing treatment-naive advanced PC, except that future prospective studies with larger sample sizes and multiple centers are required to determine its efficacy and safety.

## Introduction

Pancreatic cancer(PC) is one of the most prevalent diseases of the digestive system across the globe, with late diagnosis, rapid progression, and a poor prognosis (1, 2). With a five-year survival rate of 10%, PC is currently the fourth leading cause of cancer-related deaths (3, 4). A majority of PC patients show middle or advanced stage when they first consult a doctor owing to its insidious onset, rapid progression, and lack of typical clinical symptoms in the early stage; less than 20% of this patient group have a chance of receiving radical resection (5). Patients with inoperable metastatic PC have a 6-month median overall survival (mOS) (6). Although systemic chemotherapy remains the principal treatment for advanced PC patients to improve the quality of life and lengthen survival time (7), its overall effectiveness remains inadequate. At present, systemic chemotherapy has been found to improve median survival by 2–4 months, and it is associated with considerable toxicity (8).

Radiotherapy and immunotherapy have only made a modest improvement over the past few years in treating advanced PC patients (9, 10). Nonetheless, chemotherapy remains the first option for most advanced PC patients. Adjuvant chemotherapy with Gemcitabine (GEM) has been shown to considerably prolong disease-free survival and overall survival (OS) of PC patients (11). A novel generation of oral compound preparation from the 5-FU family, Tegafur-gimeracil-oteracil potassium (S-1) has proved in clinical tests to be effective as GEM in treating metastatic or locally advanced PC (12, 13). The 2018 edition of the guidelines of the Chinese Society of Clinical Oncology (CSCO) for Pancreatic Cancer indicates that GEM combined with an S-1 chemotherapy regimen (GS regimen) is one of the first-line treatments for advanced PC (14).

Anlotinib, as a novel oral small-molecule multi-target tyrosine kinase inhibitor, has been demonstrated to inhibit tumor growth and exert anti-tumor angiogenesis effects, effectively inhibiting PDGFR, C-KIT, FGFR, VEGFR, and other kinases that are critical to cancer progression (15, 16). In recent years, the Chinese National Medical Products Administration (NMPA) approved anlotinib for the treatment of small cell lung cancer(SCLC) (17), advanced non-small cell lung cancer (NSCLC) (16, 18), thyroid cancer (19), soft tissue sarcoma (20), and esophageal cancer (21). Many clinical trials in liver cancer, gastric cancer, colorectal cancer, kidney cancer, breast cancer, endometrial cancer, ovarian cancer, NK/T cell lymphoma, Ewing’s sarcoma, diffuse large B cell lymphoma, and other cancers are currently underway.

Considering these factors, we hypothesized that adding anlotinib to the GS regimen may improve treatment efficacy for PC, which has not previously been documented. In this view, the present work evaluated the safety and efficacy of anlotinib combined with the GS regimen (hence referred to as the A+GS regimen) in the first-line treatment of patients with metastatic or unresectable PC to better understand the therapy choices for advanced PC.

## Materials and methods

### Patient selection

This retrospective real-world study used data from patients with unresectable or metastatic PC who received GS regimen alone or in combination with anlotinib at Yueyang Central Hospital and Yueyang People’s Hospital between October 2018 and June 2022. The inclusion criteria were as follows: (1) PC diagnosis was made based on pathological examination; (2) Eastern Cooperative Oncology Group (ECOG) performance status score ≤2; (3) age ≥18 years; (4) tumor lesions are unresectable or patients are unwilling to undergo surgery for various reasons, including a small number of postoperative recurrence; (5) no other systemic therapy received before first-line chemotherapy, or neoadjuvant or adjuvant chemotherapy received with one regimen but relapsed more than 6 months after the end of last chemotherapy; (6) no obvious contraindication in using chemotherapy and antiangiogenic drugs before treatment; (7) at least one measurable lesion according to the Response Evaluation Criteria in Solid Tumors version 1.1 (RECIST 1.1); (8) projected survival time ≥3 months. The exclusion criteria were as follows: (1) Patients who received systemic treatment the last within 6 months.

Patients who received the A+GS regimen were classified in the A+GS group, whereas those who received the GS regimen were classified in the GS group. The treatment approaches were chosen with consent from patients. The study followed the Declaration of Helsinki, and because a retrospective study does not require ethics committee approval, formal informed consent from patients was waived.

### Treatment

(1) GS group: Patients received sequential chemotherapy with GEM + S-1, with GEM doses of 800-1000mg/m^2^ ivdrip d1, d8, Q3W + S-1 40-60mg po bid, d1-d14, Q3W, lasting 4 to 6 cycles, followed by S-1 monotherapy maintenance therapy. (2) A+GS group: Patients received a three-drug combination of GEM + S-1+ anlotinib, the dosage of GEM + S-1 was similar to the GS group, the anlotinib(Chia Tai Tianqing Company) dose was 8-12mg po qd, d1-d14, Q3W, administered orally before breakfast.

The dosage of oral S-1 for PC patients was calculated based on their body mass index (BMI), as follows (BMI<1.25 kg/m^2^: 40mg po bid d1-14 Q3W,1.25kg/m^2^<BMI<1.5 kg/m^2^: 50mg po bid d1-14 Q3W, BMI>1.5 kg/m^2^: 60mg po bid d1-14 Q3W), and the initial dose of anlotinib was 12mg po qd, d1-d14, Q3W. Patients were closely monitored for adverse events (AEs), and drug dose during treatment was adjusted if major AEs occurred. The dosage of anlotinib or chemotherapeutic drug was decreased appropriately if grade 3 AEs occurred, for the AEs of anlotinib, the first adjustment dosage was 10mg po qd, d1-d14, Q3W, and the second adjustment dosage was 8mg po qd, d1-d14, Q3W. Treatment was discontinued completely if 8mg was not tolerable, for the AEs of S-1 or GEM, the dose was reduced by 25% until the AEs improved to grade 0-1. Meanwhile, the current treatment was discontinued or replaced if grade 4-5 AEs occurred. In our investigation, we only recorded the incidence of grade ≥2 AEs because of the low incidence of grade 4-5 AEs. In addition, patients were allowed to receive local radiotherapy or interventional therapy. Follow-up information and clinical data were obtained using telephone or hospital records.

### Follow-up and response evaluation

Patients were regularly monitored and evaluated every 1-2 months till death or the censoring date. The size of primary tumors was measured using magnetic resonance imaging (MRI), and/or computed tomography (CT) at baseline, and then every 2-3 months during the treatment. Treatment responses were classified as complete response (CR), partial response(PR), stable disease (SD), and progressive disease (PD) using the Response Evaluation Criteria in Solid Tumors version 1.1 (RECIST 1.1) standards. The toxicity was assessed using the Common Terminology Criteria for Adverse Events version 5.0 (CTCAE5.0).

Primary outcomes were overall survival (OS) and progression-free survival(PFS). Secondary outcomes were disease control rate(DCR=CR+PR+SD), objective response rate (ORR=CR+PR), and toxic side effects. OS was defined as the time interval from the start of treatment to the date of the last follow-up or death. PFS was defined as the period between the commencement of treatment and disease progression or follow-up termination if there was no relapse or death.

### Statistical analysis

All statistical analyses were performed with SPSS 23.0 software. The Chi-square test was used to compare the clinical parameters of the two groups. The median PFS(mPFS) and mOS were estimated with the Kaplan-Meier method. Survival curves were generated using GraphPad Prism 8.0. and Cox proportional hazards regression analyses were used to identify prognostic factors influencing PFS and OS.

## Result

### Patient characteristics

Seventy-one patients (48 males and 23 females) were enrolled in this study between October 2018 and June 2022; 41 subjects were classified in the GS group, while 30 subjects were classified in the GS+A group (**Table 1**). The primary lesion developed in the head of the pancreas in 52 of these patients, and in the body or tail of the pancreas in 19 patients. Twenty-three (32.4%) patients had distant metastases, and 3 patients had tumor recurrence following previous radical resection. Simple liver metastasis occurred in 3 patients, simple lung metastasis in 2 patients, simple retroperitoneal lymph node metastasis in 1 patient, simple peritoneal metastasis in 1 patient, and simultaneous multiple metastases in 16 patients. Among them, 41 patients could not undergo surgical treatment because the tumor encircled the abdominal trunk and superior mesenteric artery or locally invaded the duodenum or liver, and 7 patients with resectable tumors were not considered for surgical treatment for various reasons. Moreover, 4 patients received local radiotherapy following first-line systemic therapy. The two groups did not differ significantly in terms of age, gender, primary tumor site, and distant metastasis state.

**Table 1:** Clinical parameters of the patients.

Characteristics	Group(n%)		*P* value
	GS group	GS+A group	
Age(year)			0.875
≤55	24(58.5%)	17(56.7%)	
>55	17(41.5%)	13(43.3%)	
Gender			0.241
Male	30(73.2%)	18(60.0%)	
Female	11(26.8%)	12(40.0%)	
Location			0.285
Head ^a^	32(78.1%)	20(66.7%)	
Tail/Body ^b^	9(21.9%)	10(33.3%)	
Distant metastasis			0.163
No	25(60.1%)	23(76.7%)	
Yes	16(39.0%)	7(23.3%)	

a The primary tumor is located in the head of the pancreas; ^b^The primary tumor is located in the tail or body of the pancreas.

### Survival outcomes

**Figure 1:**
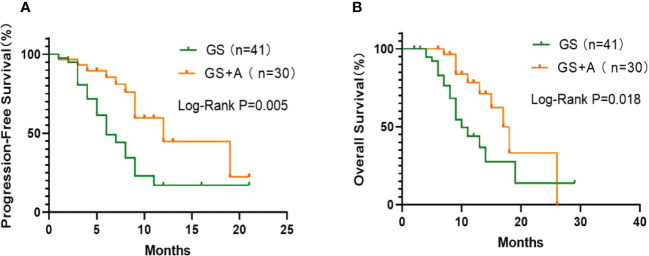
Kaplan-Meier plots: The GS+A group exhibited longer median progression‐free survival (mPFS; **(A)** and median overall survival (mOS; **(B)** than the GS.

There were no treatment-related deaths in either group during the follow-up and treatment period. At the last follow-up, 10(33.33%) and 18 (43.90%) patients in the A+GS group and the GS group, respectively, died. **Figure 1** depicts the Kaplan–Meier curves for the two groups. The A+GS group showed a longer mPFS than the GS group (12.0 months (95% confidence interval (CI), 6.0-18.0) and 6.0 months (95% CI, 3.9-8.1)), respectively (P = 0.005; **Figure 1A**). Similarly, the A+GS group had a longer mOS compared with the GS group (17.0 months (95%CI, 14.0-20.0) and 10.0 months (95% CI, 7.5-12.5)), respectively (P = 0.018; **Figure 1B**). Furthermore, the A+GS group had a longer time for disease progression and better prognosis compared with the GS group.

The A+GS group had a longer mOS (P=0.012) and mPFS(P=0.006) than the GS group in the subgroup of PC patients with head tumors. However, there was no significant difference (P>0.05) in PFS and OS between the two groups in the subgroup analysis of PC patients with tumors in the tail or body, and different distant metastasis states. **Supplementary Figure 1** depicts the comparison results and Kaplan–Meier curves for different subgroups.

### Tumor response

**Figure 2:**
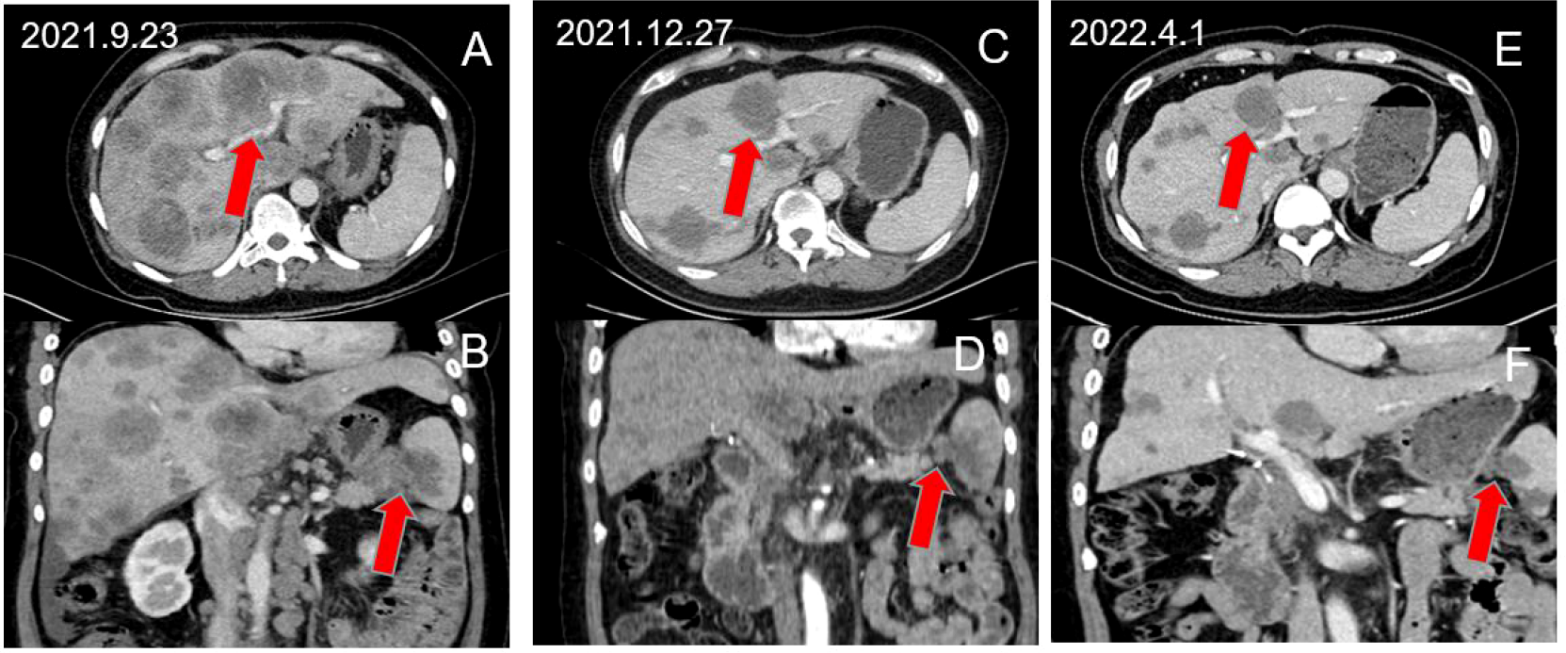
A 52-year-old female patient with advanced PC with multiple liver metastases achieved partial response after receiving A+GS regimen therapy. **(A, B)** show the pre-treatment baseline images, **(C, D)** show the images after 3 cycles of treatment, and **(E, F)** show the images after 6 cycles of treatment.

The GS+A group had 15 PR and 10 SD cases, while the GS group had 11 PR and 13 SD cases. The DCR and ORR of the A+GS group(83.3% and 50.0% respectively) were significantly higher than those of the GS group (58.5% and 26.8%, respectively) (**Table 2**). In addition, we selected a PC patient with multiple liver metastases who received the A+GS regimen as first-line treatment. **Figure 2** depicts continuous changes in CT images before and after treatment.

**Table 2:** Treatment response of the patients.

Best response	Group(n%)		*P* value
	GS+A group	GS group	
ORR	15 (50.0)	11 (26.8)	0.045
DCR	25 (83.3)	24 (58.5)	0.026
Best overall response			
CR	0	0	
PR	15 (50.0)	11 (26.8)	
SD	10 (33.3)	13 (31.7)	
PD	5 (16.7)	17 (41.5)	

ORR, Objective response rate; ORR, Disease control rate; CR, Complete response; PR, Partial response; SD, Stable disease; PD, Progressive disease.

### Adverse effects

Analysis of the adverse effects in the two groups revealed that the addition of anlotinib did not increase the incidence of adverse effects(AEs) compared with the GS regimen alone (P>0.05 for all) (**Table 3**). The chemotherapy drug dosage was adjusted for four patients due to hematological toxicity. Anlotinib dosage was adjusted for 2 patients due to hepatotoxicity; notably, the AEs resolved after dose adjustment or symptomatic supportive treatment. No grade 4-5 AEs or treatment-related deaths were recorded.

**Table 3:** Treatment-related adverse events of in patients.

Variable	Group (n%)		*P* value
	GS+A group	GS group	
Gastrointestinal reaction	12 (40.0)	14 (34.2)	0.613
Hematological toxicity	11 (36.7)	10 (24.4)	0.263
Hepatotoxicity	7 (23.3)	5 (12.2)	0.216
Hand-foot syndrome	9(30.0)	10 (24.4)	0.598
Oral mucositis	4 (13.3)	6 (14.6)	0.876

### Factors associated with OS and PFS

Prognostic indicators influencing PFS and OS were revealed with univariate and multivariate Cox regression analyses. Multivariate analysis demonstrated that distant metastasis states, tumor location, and treatment regimen were independent prognostic factors for PFS (**Table 4**), whereas tumor location and treatment regimen were independent prognostic factors for OS (**Table 5**).

**Table 4:** Univariate and Multivariate analysis of PFS.

Variable	Univariable Cox regression			Multivariable Cox regression		
	HR	95%CI	P	HR	95%CI	P
Age(≤55/>55)	0.616	0.306-1.240	0.174			
Gender(Male/Female)	0.939	0.443-1.990	0.869			
Distant metastasis(Yes/No)	2.697	1.298-5.603	0.008	3.128	1.457-6.715	0.003
Tumor location(Head/Tail or Body)	2.103	0.901-1.4.908	0.086	2.715	1.135-6.493	0.025
Regimen(GS/GS+A)	2.64	1.265-5.511	0.01	2.553	1.207-5.401	0.014

**Table 5:** Univariate and Multivariate analysis of OS.

Variable	Univariable Cox regression			Multivariable Cox regression		
	HR	95%CI	P	HR	95%CI	P
Age(≤55/>55)	1.139	0.533-2.432	0.737			
Gender(Male/Female)	0.832	0.349-1.985	0.679			
Distant metastasis(Yes/No)	1.436	0.301-1.609	0.397	2.076	0.856-5.031	0.106
Tumor location(Head/Tail or Body)	3.018	1.125-8.097	0.028	3.368	1.229-9.231	0.018
Regimen(GS/GS+A)	2.46	1.120-5.404	0.025	2.556	1.139-5.733	0.023

## Discussion

The NCCN and CSCO guidelines for pancreatic adenocarcinoma (14, 22), for unresectable locally advanced PC or patients with distant metastasis, recommend GS regimen, GA regimen (GEM, albumin-bound paclitaxel), GX regimen (GEM, Capecitabine), FOLFRINOX (oxaliplatin, fluorouracil, irinotecan, and leucovorin), among others, as the first-line treatment options. However, the overall treatment efficacy remains unsatisfactory. In this view, more relevant therapeutic modalities that can improve the prognosis of patients with advanced PC are urgently needed. To the best of our knowledge, this was the first study comparing A+GS regimen vs GS regimen alone in managing advanced PC.

The preliminary findings of this retrospective real-world investigation demonstrated that the A+GS group had better ORR and DCR, longer mOS, and mPFS than the GS group in the treatment of patients with advanced PC. Additionally, there was no discernible increase in AEs between the A+GS group and the GS group, indicating a satisfactory safety profile. These data demonstrate a novel therapeutic approach that may benefit patients with treatment-naive advanced PC in terms of survival and efficacy.

According to recent evidence, neither immunotherapy nor targeted therapy, two recent revolutions in cancer treatment, have produced statistically significant positive results in PC treatment (23). Immunotherapeutic interventions and targeted therapies are not currently the primary treatment options for PC (9, 24, 25), and the benefit of radiotherapy is also insufficient (26). Of note, GEM has been the first-line chemotherapy regimen for PC patients with the locally advanced or metastatic disease since 1997, but the mOS of PC patients treated with single-agent GEM was only 5.7 months (27). Several combinations of GEM with biological agents and cytotoxic agents have been investigated, but a majority have not significantly improved prognosis as compared with GEM alone (28–31). Although GEM is widely accepted as the first-line treatment option for advanced PC, overcoming GEM resistance remains a significant challenge for pancreatic cancer patients.

S-1 is an oral chemotherapeutic drug that is well-tolerated and simple to administer in clinical practice. S-1 outperformed GEM in terms of OS in the GEST Phase III clinical trial research, with an mOS of 8.8 months for GEM and 9.7 months for S-1 in the treatment of patients with locally advanced or metastatic PC (12). S-1 monotherapy and GS regimen have been listed as first-line chemotherapy options for unresectable locally advanced or metastatic PC in the 2018 CSCO guidelines (14).

EGFR overexpression was reported in approximately 30% to 89% of PC patients (32). GEM combined with erlotinib, an EGFR tyrosine kinase inhibitor, was found to be more effective than GEM alone in both mPFS and mOS in metastatic or locally advanced PC in phase III randomized controlled clinical study. However, the mOS was only extended by 0.33 months (6.24 months vs 5.91 months) and the mPFS was only extended by 0.20 months (3.75 months vs 3.55 months) (33). Furthermore, because the combination of GEM and erlotinib has limitations in prolonging PFS and OS in patients with metastatic or locally advanced PC compared to GEM monotherapy, it is necessary to investigate a novel small molecule tyrosine-kinase inhibitor drug in combination with chemotherapy for PC.

Anlotinib is a novel oral small-molecule tyrosine kinase inhibitor with multi-targets. Anlotinib, unlike other tyrosine kinase inhibitors such as sunitinib and sorafenib, can effectively inhibit multiple targets, among them FGFR, PDGFR, VEGFR, C-Kit, and other kinases (34). Since its introduction as a broad-spectrum anti-tumor-targeted drug, anlotinib has made significant progress in the treatment of cancers and has played a role in a wide range of malignancies (34–37). Zhang et al. discovered that anlotinib inhibited PC cell proliferation while inducing apoptosis (38). Yang et al. demonstrated that anlotinib killed PC cells both *in vitro* and *in vivo* (39). Moreover, several case reports show that anlotinib improved the prognosis of patients with advanced PC (40–42). A retrospective study of 33 patients with advanced PC (17 patients received anlotinib combined with GA regimen (GEM, albumin-bound paclitaxel) and 16 patients received GA regimen alone revealed that the anlotinib combination GA regimen group had significantly improved mOS (9.0months vs 6.0 months, P = 0.006) and mPFS(5.0months vs 2.7months, P = 0.022) when compared with the GA regimen group alone (43). In this view, anlotinib is expected to achieve a therapeutic advantage in treating metastatic and locally advanced PC.

The present investigation revealed that the A+GS group had a longer mOS and mPFS than the GS group in the subgroup of PC patients with tumors in the head. However, in the other subgroups, no significant difference in PFS and OS was reported between the two groups, which could be attributed to the smaller sample size. Furthermore, our multivariate analysis revealed that distant metastasis and tumor location are both independent risk factors for PFS and OS, respectively. Similarly, previous research found a link between distant metastasis and prognosis (5). Although there is controversy regarding whether cancer location is a prognostic factor in PC (44), we could not extensively explore this phenomenon due to the small sample size. Previous studies revealed that the adverse reactions of anlotinib are relatively mild, and the proportion of patients whose dose was reduced or discontinued due to adverse reactions was low than in group GS (45). In our investigation, the most common AEs in the two groups were in most cases mild and manageable, including hematological toxicity, hepatotoxicity, and gastrointestinal reactions. Furthermore, the incidence of AEs was not significantly different between the two groups, demonstrating that A+GS regimen therapy is clinically safe and feasible. Our findings strongly demonstrate that anlotinib improves the efficacy of the GS regimen. Although individual differences may influence patient prognosis, the short-term efficacy, based on PFS and tumor response rate, that the present work fully evaluated was not affected by subsequent treatment. These data could accurately represent the clinical efficacy of A+GS regimen therapy. As such, we will provide more data on efficacy and safety in the future.

While the present investigation is the first to report preliminary clinical results of anlotinib combined with GS regimen for advanced PC, providing evidence for future prospective clinical trials, a few limitations cannot be ignored. First, this was a retrospective real-world study conducted in China with small sample size and inevitable potential bias. Second, although this is the largest study reported so far, the number of PC patients in the A+GS regimen therapy group remained small. Future prospective multicenter randomized clinical trials are warranted to validate these findings.

## Conclusions

The A+GS regimen therapy is of great promise in managing treatment-naive advanced PC. Our data provide a theoretical foundation for further investigation of the A+GS regimen therapy for advanced PC. The efficacy of the A+GS regimen therapy warrants further validation with more prospective studies with larger sample sizes and multiple centers.

## Data availability statement

The original contributions presented in the study are included in the article/**Supplementary Material**. Further inquiries can be directed to the corresponding authors.

## Ethics statement

Ethical review and approval was not required for the study on human participants in accordance with the local legislation and institutional requirements. Written informed consent for participation was not required for this study in accordance with the national legislation and the institutional requirements.

## Author contributions

YS and DZ contributed to the idea and design. GZ performed the research and edited the manuscript. JH, SD, JW and CZ organized and data analyzed results. FW, SL, FF, ES, QZ, PL and MX contributed to the manuscript writing and revision. All authors approved the final manuscript. All authors contributed to the article and approved the submitted version.
