# Supplementary material for: A real-world study of anlotinib combined with GS regimen as first-line treatment for advanced pancreatic cancer
Source: Front Endocrinol (Lausanne). 2023 Jan 20;14:1110624. doi: 10.3389/fendo.2023.1110624 (PMC9897321; doi:10.3389/fendo.2023.1110624)

Supplementary Figure1. Kaplan-Meier plots: Overall survival curves for the patients with tumor location(A) and distant metastases(B), Progression-free survival curves for the patients with tumor location(C) and distant metastases(D).

**A**


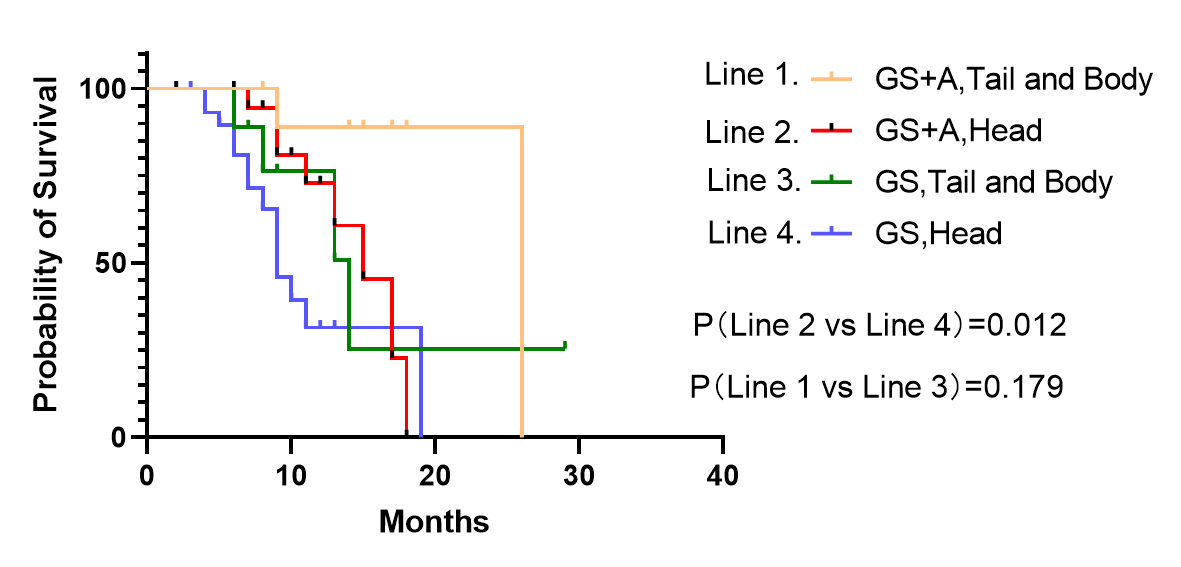


**B**


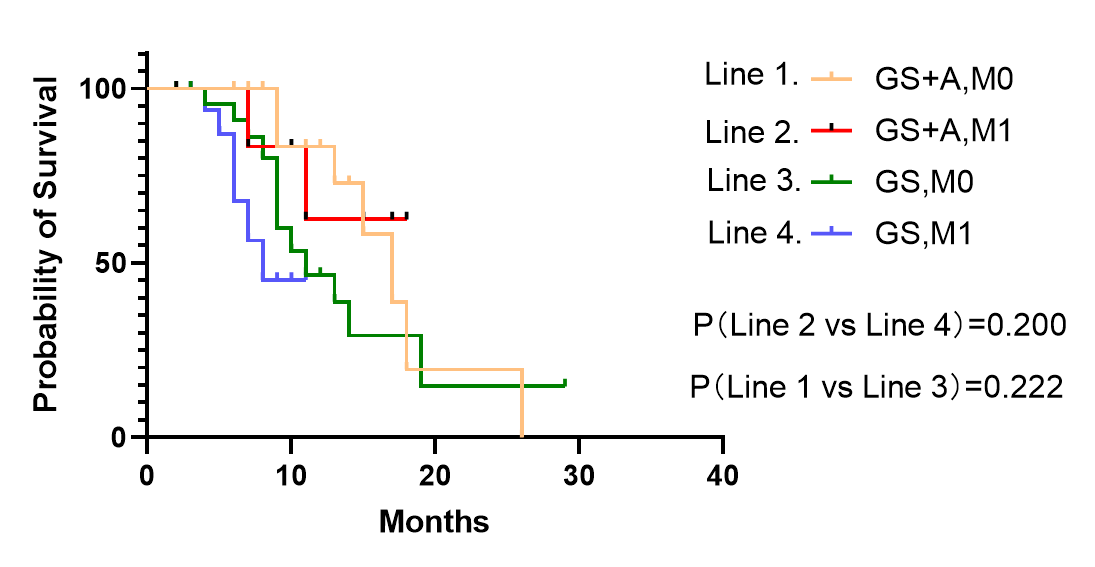


**C**


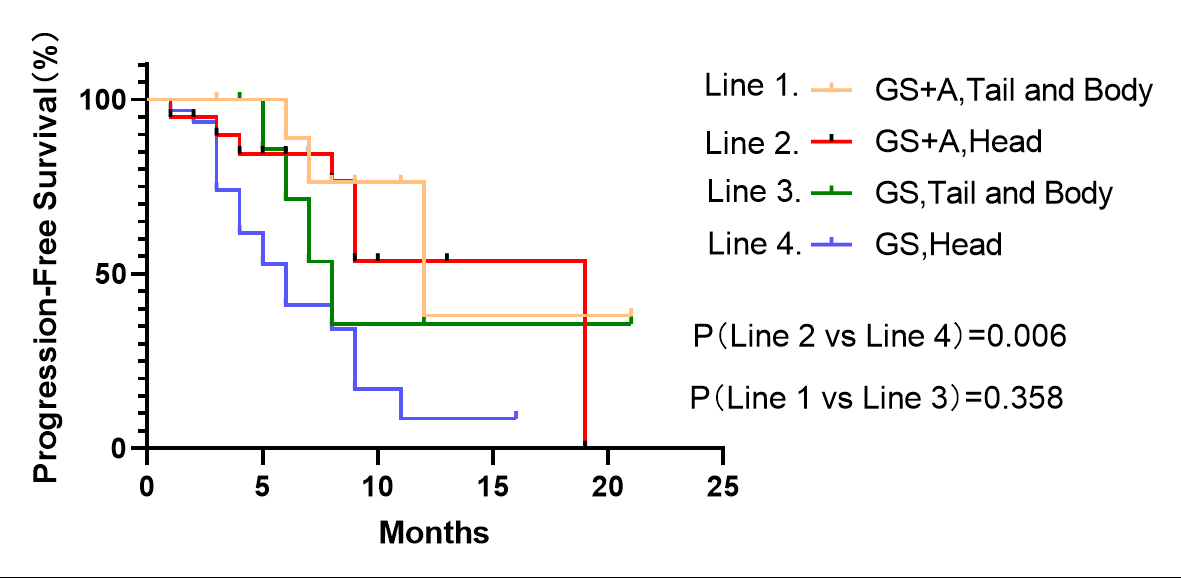


D


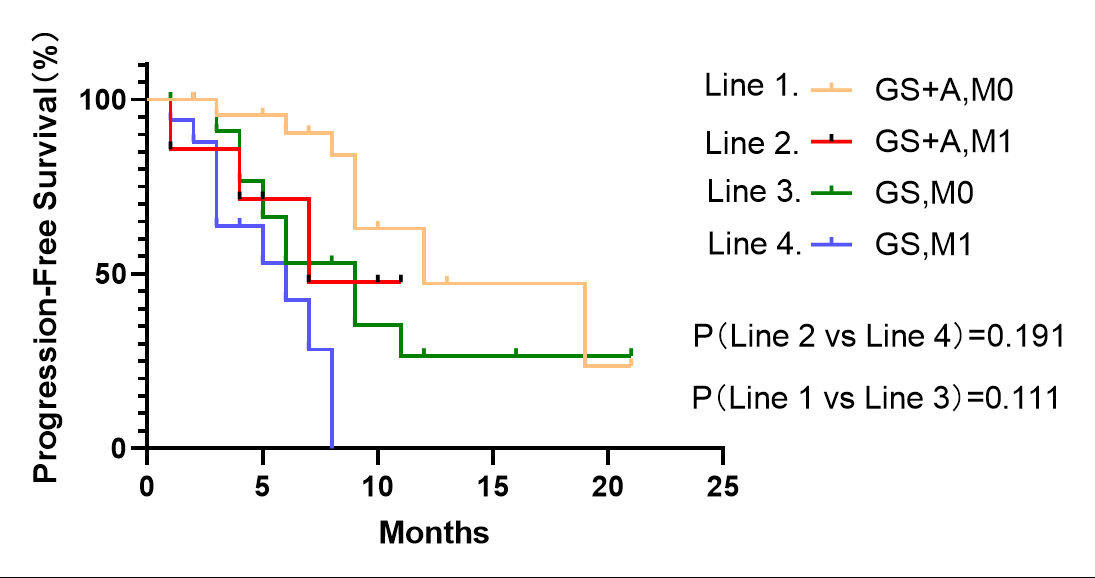

Supplement: Supplementary file 1 [file DataSheet_1.doc]
